# Influence of dietary canola oil and palm oil blend and refrigerated storage on fatty acids, myofibrillar proteins, chemical composition, antioxidant profile and quality attributes of *semimembranosus* muscle in goats

**DOI:** 10.1186/s40104-015-0050-z

**Published:** 2015-12-03

**Authors:** Kazeem D. Adeyemi, Azad B. Sabow, Rafiat M. Shittu, Roselina Karim, Awis Q. Sazili

**Affiliations:** Department of Animal Science, Faculty of Agriculture, 43400 UPM, Serdang, Selangor Malaysia; Halal Products Research Institute, 43400 UPM, Serdang, Selangor Malaysia; Laboratory of Animal Production, Institute of Tropical Agriculture, 43400 UPM, Serdang, Selangor Malaysia; Department of Food Technology, Faculty of Food Science and Technology, Universiti Putra Malaysia, 43400 UPM, Serdang, Selangor Malaysia; Department of Animal Production, University of Ilorin, PMB 1515, Ilorin, Nigeria; Department of Animal Resource, University of Salahaddin, Erbil, Kurdistan Region Iraq

**Keywords:** Actin, Cholesterol, Lipid oxidation, Meat quality, Myosin, Protein oxidation

## Abstract

**Background:**

Improving the unsaturated fatty acid content of ruminant meat is essential due to the generally saturated nature of fatty acids in ruminant meat and the negative effects this can have on human health. Nonetheless, enhancing the unsaturated fatty acid content of ruminant meat can have adverse effects on the shelf life and quality attributes of the meat. This study assessed the effects of dietary 80 % canola oil and 20 % palm oil blend (CPOB) on fatty acid composition, antioxidants, oxidative spoilage, cholesterol and physicochemical properties of *semimembranosus* (SM) muscle from goats. Twenty four Boer bucks were randomly assigned to diets containing on dry matter basis 0, 4 and 8 % CPOB, fed for 100 d and slaughtered. The carcasses were subjected to a 7 d postmortem refrigerated storage. All analyses were conducted on the SM muscle.

**Results:**

Diet had no effect (*P >* 0.05) on the concentration of free thiol and carbonyl and the band intensity of myosin heavy chain, actin and troponin T. The muscle glycogen, pH, water holding capacity, tenderness, glutathione peroxidase (GPX) activity, total carotenoid, δ-tocopherol, cholesterol and proximate composition did not differ (*P* > 0.05) between diets. The SM muscle from goats fed 4 and 8 % CPOB had lower (*P <* 0.05) concentration of C14:0 and C16:0 and higher (*P <* 0.05) concentration of C18:1 *trans*-11, C18:1ω-9, C18:3ω-3, C20:5ω-3 and C22:5ω-3 than the SM muscle from the control goats. Dietary CPOB increased (*P <* 0.05) the concentration of α and γ tocopherol and meat redness (a*) on d 1 and 4 postmortem. Regardless of diet, antioxidant vitamins, and shear force decreased (*P <* 0.05) while drip loss, lipid and protein oxidation increased (*P <* 0.05) as postmortem storage progressed.

**Conclusion:**

Results evince that dietary CPOB can be used as a management tool to enhance the beneficial fatty acids and antioxidant contents of chevon without deleterious effects on its physicochemical properties and shelf life.

## Background

Ruminant meat is an important source of protein and essential nutrients with enhanced bioavailability compared with other dietary sources [1] and it is valued in many cultural culinary traditions. However, in recent times, consumers are cautioned against the consumption of ruminant meat because the fat it contains is more highly saturated and this was believed to be a factor predisposing to chronic diseases [2–4]. Nonetheless, due to the nutritionally dense nature of ruminant meat, lowering its consumption could have inadvertent health and nutritional consequences [5]. Thus, enhancing the beneficial unsaturated fatty acids (UFA) and/or reducing the saturated fatty acids (SFA) in ruminant meat is a continued research endeavor [6]. One major way of altering the lipid composition of ruminant meat is dietary supplementation of oils [7, 8]. However, dietary manipulation of muscle fatty acids could predispose the meat to lipid and protein oxidation [9] which are major causes of non-microbial quality deterioration in foods [10, 11]. Lipid and protein oxidation can instigate loss of nutritional and sensory quality of meat [9, 12] and lower its shelf life [9, 13]. In addition, lipid and protein oxidation can generate toxic compounds that can have adverse effects on human health [14]. Hence, enhancing the UFA content of ruminant meat warrants the protection of such meat from oxidative spoilage via the use of antioxidants. Although, synthetic antioxidants are effective in delaying lipid and protein oxidation, they have been implicated in pathogenicity, toxicity and carcinogenic effects on animals and humans [15]. This necessitates the use of natural antioxidants whose efficacy in curbing lipid and protein oxidation has been espoused [16–18]. Aside the potency of natural antioxidants in curbing nutritional loss in food and to extend its shelf life, their roles in preventing chronic health related problems in humans have been documented [19, 20]. The use of antioxidant rich oil has been suggested as an economically feasible way of curbing lipid and protein oxidation and a viable means of promoting antioxidants in human diet [21]. Thus, due to the antioxidant contents and fatty acid profile of canola oil [22] and palm oil [23], it was hypothesized that a blend of canola oil and palm oil will enhance the lipid profile, physicochemical properties and oxidative stability of chevon. The objective of this study was to determine the effect of graded levels of dietary 80 % canola oil and 20 % palm oil blend (CPOB) and refrigerated storage on fatty acid composition, cholesterol content, antioxidant status, meat quality and oxidative stability of lipid and myofibrillar proteins in *semimembranosus* muscle from goats.

## Materials and methods

### Animal welfare

This study was conducted following the guidelines approved by the Universiti Putra Malaysia Institutional Animal Care and Use Committee. The animals’ health and welfare were monitored by qualified veterinarians who were members of the research team.

### Animals and diets

The feeding trial was conducted at ar-Raudhah Pty. Ltd. Kuang, Selangor, Malaysia. The geographical coordinates of Kuang are 3° 16' 0" North, 101° 35' 0" East. Annual rainfall was about 2,670 mm. During the trial, the ambient temperature ranged from 26-33 °C while relative humidity ranged from 81-85 %. Twenty four entire Boer bucks of 4-5 months old, having initial body weight of 20.54 ± 0.47 kg were used for the study. Each animal was individually housed in wooden slatted floor pen equipped with feeding and drinking facilities. The animals were randomly assigned to three diets containing 0, 4 and 8 % CPOB on DM basis and fed for 100 d following 14 d of adaptation. All diets were formulated to meet the nutritional requirements of growing goats following the recommendation of NRC [24]. The basal diet was made up of a 50:50, forage to concentrate ratio. The forage portion was oil palm fronds. The concentrate consisted of 22 % corn grain, 17 % soybean meal, 7.5 % palm kernel cake and 2 % rice bran. Each of limestone, salt and mineral-vitamin premix accounted for 0.5 % of the mixed diet. Ingredients in the concentrate portion were adjusted to make the diets isocaloric and isonitrogenous. The chemical, fatty acid and antioxidant composition of the diets are presented in Table 1.

**Table 1:** Chemical, fatty acid and antioxidant composition of dietary treatments

	Level of CPOB^a^, %		
Chemical composition (% DM)	0	4	8
Dry matter	67.70	67.90	68.07
Crude Protein	14.27	14.37	14.39
Ether extract	2.30	6.35	11.11
Organic matter	93.16	93.42	93.55
Nitrogen free extract	16.56	13.97	12.45
Acid detergent fibre	35.04	33.28	32.52
Neutral detergent fibre	63.52	62.67	62.06
Metabolizable energy, MJ/Kg DM	11.59	11.61	11.62
Ca	1.02	1.05	1.04
P	0.52	0.54	0.54
Fatty acids, g/kg DM			
C12:0	0.01	0.03	0.04
C14:0	0.53	0.51	0.51
C16:0	2.79	5.98	7.78
C16:1ω-7	0.08	0.11	0.15
C18:0	0.56	1.12	1.43
C18:1ω-9	3.82	14.87	26.32
C18:2ω-6	7.05	11.87	12.06
C18:3ω-3	1.06	2.61	4.13
ω-6:ω-3	6.66	4.54	2.92
Total FA	15.83	37.09	52.27
Total carotenoid, mg/kg	14.81	16.71	19.86
α-tocopherol, mg/kg	101.12	112.47	123.21
γ-tocopherol, mg/kg	10.22	34.55	49.17
δ-tocopherol, mg/kg	1.21	3.45	5.93

^a^80 % canola oil and 20 % palm oil blend. OPF^2^ = oil palm frond. ω-6:ω-3 = (C18:2ω-6÷C18:3ω-3)

### Slaughtering procedure and muscle sampling

The animals were fasted for 12 h with free access to water, weighed and slaughtered according to the halal procedure as outlined in MS1500:2009 [24]. After dressing, the carcasses were subjected to a 7 d postmortem refrigerated (4 °C) aerobic storage. On d 0, 35 g of *Semimembranosus* (SM) muscle was sampled at the posterior face of the left hind limb and divided into three portions. The first portion (10 g) was snap frozen in liquid nitrogen, stored at -80 °C and assigned for the determination of fatty acids, cholesterol, lipid and protein oxidation, myofibrillar protein, antioxidants, glycogen and pH at d 0. The second portion (10 g) was weighed, vacuum packed and stored at 4 °C for the determination of drip loss on d 1, 4 and 7 postmortem. The third portion (15 g) was assigned for the determination of color, cooking loss and shear force. The remaining SM samples were left on the carcass intact and removed after 1, 4 and 7 d of storage. After the completion of each ageing period, samples (25 g each) were dissected from the carcasses and divided into two portions. The first portion (10 g) was snap frozen in liquid nitrogen and assigned for the determination of lipid and protein oxidation, myofibrillar protein, antioxidants, glycogen and pH while the remaining 15 g portion was used for color, cooking loss and shear force analyses.

### Determination of muscle pH

The pH of *semimembranosus* muscle at 45 min and 24 h postmortem was read using a pre calibrated portable pH meter (Mettler Toledo, AG 8603, Switzerland). One gram of pulverized (crushed with porcelain mortar and pestle with continuous flushing with liquid nitrogen) muscle sample was homogenized for 30 s in 5 mL of cold deionized water in the presence of 5 mmol/L sodium iodoacetate (Merck Schuchardt OHG, Germany) to prevent further glycolysis. The pH of the resultant homogenate was read using the electrode attached to the pH meter.

### Determination of muscle glycogen

The glycogen content in the pulverized *semimembranosus* muscles (200 mg each) was determined using EnzyChrom™ Glycogen Assay kit (Cat# E2GN-100, BioAssays, USA) following the manufacturer’s procedure. In the assay, glycogen was hydrolyzed to glucose by glucoamylase enzyme which was then oxidized to produce a product which reacted with OxiRed probe. The color generated from this reaction was measured at 570 nm wavelength using an auto microplate reader (infinite M200, Tecan, Austria).

### Determination of lipid oxidation

Lipid oxidation was measured as 2-thiobarbituric acid reactive substances (TBARS) using QuantiChrom™ TBARS Assay Kit (DTBA-100, BioAssay Systems, USA) following the manufacturer’s description of the colorimetric protocol. The assay was based on the reaction of thiobarbituric acid reactive substance (TBARS) with thiobarbituric acid (TBA) to form a pink colored product whose absorbance was read at 535 nm by a microplate reader (infinite M200, Tecan, Austria).

### Determination of glutathione peroxidase (GPx) activity

The GPx activity was measured with the aid of EnzyChromTM Glutathione Peroxidase Assay (Kit EGPX-100, BioAssay Systems, USA) following the manufacturer’s procedure. The assay measured the consumption of nicotinamide adenine dinucleotide phosphate (NADPH) in the enzyme coupled reactions by recording the decrease in absorbance at 340 nm. The GPx was expressed as nmoles NADPH oxidized /min/mg protein.

### Determination of free thiol content

The free thiol contents was quantified according to Elman’s method using 2,2-dithiobis (5-nitropyridine) DTNP [25]. The thiol concentration was measured using a spectrophotometer (Spectronic instruments, USA) at 386 nm and was calculated using an absorption coefficient of 14 mmol/L/cm. The results were expressed as nanomoles of free thiols per milligram of protein.

### Determination of carbonyl content

The carbonyl content in muscles was determined using Cayman protein carbonyl colorimetric assay kit (10005020) following the manufacturer’s procedure. Results were expressed as nmol/mg protein.

### Extraction of myofibrillar proteins

Myofibrillar proteins were isolated using an extraction buffer containing 150 mmol/L NaCl, 25 mmol/L KCl, 3 mmol/L MgCl_2_, 4 mmol/L EDTA at pH 6.5 as described by Morzel et al*.* [26]. The total protein concentration of the samples was determined by the method of Bradford [27] using Protein Assay Kit (II 500-0002, Bio-Rad, USA). Bovine Serum Albumen (BSA) was used to prepare the protein standards.

### Sodium dodecyl sulphate polyacrylamide gel electrophoresis (SDS-PAGE)

Myofibrillar proteins were incubated for 10 min at 90 °C in a sample buffer containing 30 % (v/v) glycerol, 5 % (v/v) ß-mercaptoethanol, 2.3 % (w/v) SDS, 62.5 mmol/L Tris–HCl (pH 6.8) and 0.05 % (w/v) bromophenol blue at a ratio of 1:1(v/v). One dimensional SDS-PAGE was performed according to the method of Laemmli [28] using polyacrylamide gels of 8 cm × 5.5 cm (length × width) and 0.8 mm thickness. Twelve percent resolving gels were prepared for actin and troponin T and 5 % resolving gels were prepared for myosin heavy chain (MHC). The resolving gels were over-laid with 4 % stacking gel solution and kept overnight at 4 °C to allow complete polymerization. The protein load was adjusted to 30 μg per lane. Proteins were separated in running buffer containing 0.025 mol/L Tris base, 0.192 mol/L glycine, 0.1 % SDS at pH 8.3 under constant voltage of 120 V and 400 mA for 90 min, during which the tracking dye reached the bottom of the gel. Protein bands were stained with 0.05 % Coomassie blue staining solution for 60 min and destained with destaining solution for 30 min [29]. The bands of myofibrillar proteins were visualized using GS-800 Calibrated Imaging Densitometer (Bio-Rad, USA).

### Western-blotting

The fractionated proteins that were initially separated from the samples based on their molecular weight through gel electrophoresis were transferred from the gel onto polyvinylidene difluoride (PVDF) membranes using Trans-Blot® SD semi-dry transfer system cell (Bio-Rad, USA). Myosin heavy chain (MHC) was transferred at a constant amperage of 250 mA and a voltage limit of 25 V for 135 min per gel while actin and troponin T were transferred at the same amperage and voltage as MHC for 45 min per gel. After transfer, membranes were blocked for 3 h at room temperature in blocking solution (5 % BSA in TBST buffer (100 mmol/L Tris-HCl, 150 mmol/L NaCl and 0.05 % Tween 20)). Blots were washed thrice (10 min per wash) in TBST buffer and incubated overnight at room temperature with the primary antibody which was diluted at 1: 500 in TBST containing 3 % BSA. Monoclonal Anti-Myosin (Skeletal, Fast, produced in mouse; Cat no. # M4276, Sigma- Aldrich, USA), Monoclonal Anti-Myosin (Skeletal, Slow, produced in mouse; Cat no. # M842, Sigma- Aldrich, USA), monoclonal Anti actin antibody (produced in rabbit; Cat no. # A2066 227, Sigma- Aldrich, USA) and monoclonal anti-troponin-T antibody (produced in mouse; Cat no. T6277, Sigma- Aldrich, USA) were the primary antibodies used for myosin heavy chain (fast), myosin heavy chain (slow), actin and troponin T respectively. Subsequently, the membranes were incubated with secondary antibody [anti- mouse IgG (whole molecule) – conjugated with peroxidase, antibody developed in rabbit; Cat no. # A9044, Sigma- Aldrich, USA], diluted (1:10,000) in 3 % BSA in TBST buffer for 90 min at room temperature. Thereafter, the membranes were washed thrice with TBST buffer. The blocked membranes were detected using a DAB substrate kit Code: E885 (DAB SUBSTRATE SYSTEM (aMReSCO®, Solon, DH, USA)). Myosin heavy chain, actin and troponin band intensities were measured using GS-800 Calibrated Imaging Densitometer (Bio-Rad, USA) followed by the quantification of the bands intensity using Quantify One® software. The concentration of each myofibrillar protein was estimated [30] using the following formula:

$$ \mathrm{Concentration}\ \left(\mathrm{mg}\right) = \mathrm{Relative}\ \mathrm{intensity} \times \mathrm{Area} $$

### Determination of drip loss and cooking loss

Drip and cooking losses were measured as described by Sabow et al. [30]. For drip loss, the fresh SM muscles obtained from the carcasses on 0 d were individually weighed (approximately 15 g) and recorded as initial weight (W1). The weighed samples were placed into polyethylene plastic bags, labeled, vacuum packaged and stored at 4 °C. After 1, 4 and 7 d postmortem, the samples were removed from the bags, gently blotted dried, weighed and recorded as W_2._ Drip loss was calculated and expressed as the percentage of difference between initial and final weight of sample after storage divide by the initial weight of sample.

$$ \mathrm{Drip}\ \mathrm{loss}\ \left(\%\right) = \left[\left(\mathrm{W}1 - \mathrm{W}2\right) \div \mathrm{W}1\right] \times 100 $$

For cooking loss, the SM muscle removed from each carcass on d 1, 4 and 7 was weighed and recorded as initial weight (W1), placed in water-impermeable polyethylene bags and vacuum packed. The samples were cooked in a pre-heated water bath set at 80 °C. When the internal temperature of the samples reached 80 °C [monitored using a stabbing temperature probe (HI 145-00 thermometer, HANNA® instruments, USA) inserted into the geometric center of the sample], the cooked samples were removed from the water bath, equilibrated to room temperature and removed from the bags, blotted dried without squeezing, and reweighed (W2). The percentage cooking loss was calculated using the following equation:

$$ \mathrm{Cooking}\ \mathrm{loss}\ \left(\%\right) = \left[\left(\mathrm{W}1 - \mathrm{W}2\right) \div \mathrm{W}1\right] \times 100 $$

### Determination of shear force

The samples used for cooking loss were used to determine the shear force values. Meat textural assessment was conducted using the TA.HD plus® texture analyzer (Stable Micro System, Surrey, UK) equipped with a Volodkevitch bite jaw. The equipment was calibrated at 5 kg for weight, 10 mm return distance for height and the blade speed was set at 10 mm/s. The sample preparation was conducted following the procedure of Sazili et al. [31]. From each sample, 3 replicate blocks (1 cm height × 1 cm width × 2 cm length) were cut parallel to the direction of the muscle fibers and each block was sheared in the center perpendicular to the longitudinal direction of the fibers. Shear force was reported as the average peak positive force values for all blocks of individual sample.

### Determination of color coordinates

Meat color coordinates were determined using Color Flex spectrophotometer (Hunter Lab Reston, VA, USA) based on the International Commission on Illumination (CIE) Lab-values (also known as lightness (L*), Redness (a*) and yellowness (b*) with D65 illuminant and 10° standard observer, tristimulus values (X,Y,Z) and reflectance at specific wavelength (400-700) nm. The device was calibrated against black and white reference tiles prior to use. The SM muscle samples of approximately 15 mm thickness obtained from the carcasses on 1, 4 and 7 d postmortem were bloomed for 30 min. The bloomed surface was placed at the base of the color flex cup. For each sample, a total of three readings for L*, a* and b* values were recorded and then averaged [32].

### Determination of total carotenoids

The carotenoid content in feed and pulverized meat samples was extracted and quantified following the method of Okonkwo [33]. Two gram of each sample was homogenized with 10 mL acetone. The contents were stirred for 30 min and two 5 mL aliquot of acetone was used to rinse the flask and re-extract the residue. The extracts were pooled and 1 mL of deionized water was added. The mixture was transferred into 5 mL n-hexane and centrifuged at 3,000 g for 10 min. The absorbance of the hexane layer was read at 450 nm using spectrophotometer (Secomam, Domont, France). The carotenoid content was calculated by the following formula

$$ \mathrm{Concentration}\ \left(\mu \mathrm{g}/\mathrm{g}\right) = \left(\mathrm{A} \times \mathrm{V} \times 1{0}^4\right)/\left(2592 \times \mathrm{W}\right) $$

Where A = absorbance

V = Volume of n-hexane (mL)

W = Sample weight (g)

### Determination of cholesterol content

Cholesterol was determined using the method of Rudel and Morris [34]. One gram of pulverized sample was homogenized with 3 mL 95 % ethanol and 2 mL 50 % potassium hydroxide solution. The homogenates were incubated in water bath at 60 °C for 10 min and cooled at room temperature. Thereafter, 5 mL hexane and 3 mL deionized water was added to the homogenates, mixed for 20 s and allow to settle at room temperature until complete phase separation. About 2.5 mL of the upper phase (hexane layer) was transferred into a clean glass tube and evaporated to dryness under nitrogen gas flow at 60 °C. The residues was re-suspended with 4 mL of o-phthalaldehyde reagent and kept at room temperature for 10 min. Thereafter, 2 mL of concentrated sulfuric acid was slowly added to the mixture, mixed gently, and allow to stand for 10 min at room temperature. Cholesterol standards (Sigma L-4646) were prepared to make final concentrations of 0, 10, 20, 30, 40, 50, 60, 70, 80, 90, and 100 μg/ mL cholesterol. The absorbance of the standards and samples were read at 550 nm using a spectrophotometer (Secomam, Domont, France). An absorbance-concentration calibration curve was plotted for the cholesterol standards and the cholesterol concentration of the sample was estimated from the equation.

### Determination of tocopherol content

Extraction of tocopherol from feed and tissue samples was done in accordance to the method of Kamal-eldin et al. [35]. Quantification of tocopherol contents was done with Agilent 1200 series HPLC. The column used was C_30_ YMC™ carotenoid (250 mm  ×  4.6 mm. i.d, 5 μm) (YMC, USA). An isocratic mobile phase made up of 99 % n-hexane and 1 % Isopropanol was used. The flow rate was 0.5 mL/min and the injection volume was 20 μL. The UV detection was monitored at 295 nm. The isomers of tocopherol were quantified by comparing the peak area of sample with those of tocopherols standards in the HPLC controller software.

### Determination of fatty acids (FA)

The total fatty acids in muscle and feed samples was extracted in chloroform: methanol (2:1, v/v) mixture following the method of Rajion et al. [36]. The extracted FA were transmethylated to their fatty acid methyl esters (FAME) using 0.66 N KOH in methanol and 14 % methanolic boron trifluoride (BF_3_) (Sigma Chemical Co., St. Louis, MO, USA) according to the methods of AOAC [37]. Heneicosanoic acid was used as internal standard. The FAME was separated in a gas chromatograph (Model 6890 Agilent Technologies, USA) equipped with a FID detector and a splitless injector. The column used was fused silica capillary (Supelco SP-2560, 100 m, 0.25 mm ID, 0.20 mm film thickness). High purity nitrogen was used as the carrier gas at 40 ml/min. Compressed air and high purity hydrogen were used for the flame ionization detector in the chromatograph. To facilitate optimal separation, the oven temperature was set at 100 °C for 2 min and warmed to 170 °C at 10 °C /min, held for 2 min, warmed to 230 °C at 5 °C /min and then held for 20 min. A reference standard (mix C4–C24 methyl esters; Sigma-Aldrich, Inc., St. Louis, MO, USA) and CLA standard mix (CLA *cis*-9, *trans*-11 and CLA *trans*-10, *cis*-12, Sigma-Aldrich, Inc., St. Louis, MO, USA) were used to determine recoveries and correction factors for the determination of individual FA composition.

### Determination of chemical composition

Samples of semimembranosus muscle obtained from the carcasses on d 0 postmortem were trimmed free of external fat and epimyseal connective tissue, pulverized in liquid nitrogen and assessed for moisture, protein, crude fat and ash following the method of AOAC [37]. The proximate composition of the diets was determined following the method of AOAC [37] while the acid detergent fibre (ADF) and neutral detergent fibre (NDF) were determined following the guidelines of Van Soest et al. [38].

### Statistical analysis

The experiment followed a completely randomized design. Data obtained for fatty acids, cholesterol and chemical composition was subjected to the generalized linear model (GLM) procedure of SAS [39] in which the parameters were fitted as dependent variables while diet was fitted as fixed effect. Polynomial contrasts (linear and quadratic effects) were tested with coefficients estimated based on the level of dietary oil. Data obtained for antioxidants, physico-chemical properties and lipid and protein oxidation was analyzed using the GLM procedure of SAS [39] in which dietary treatments, postmortem storage days and interaction between dietary treatments and postmortem storage were fitted as fixed effects in a repeated measure analysis of variance. Means were separated using Tukey HSD test at significant level of *P <* 0.05.

## Results

The effect of dietary CPOB on the FA composition and cholesterol content of *semimembranosus* muscle from goats is shown in Table 2. The major fatty acids were C16:0, C18:0 and C18:1ω-9. The SM muscle from goats fed 4 and 8 % CPOB had lower (*P <* 0.05) concentration of C14:0, C16:0 and ω6/ω3 than the control goats. The concentration of C18:1 *trans*-11; C18:1ω-9, C18:3ω-3, C20:5ω-3, C22:5ω-3 and total ω-3 FA increased (*P <* 0.05) as the level of CPOB increase in diet. No significant differences (*P* > 0.05) were found for the proportions of total SFA and total MUFA between the treatments. Diet did not influence (*P* > 0.05) the PUFA/SFA ratio and cholesterol content (Table 2) and chemical composition (Table 3) of SM muscle from goats.

**Table 2:** Effect of dietary treatments on fatty acid composition (mg/100 g wet muscle) and cholesterol (mg/100 g wet muscle) of *semimembranosus* muscle in goats

	Level of CPOB^a^, %				*P* value		
Fatty acids	0	4	8	SE	Overall	Linear	Quadratic
C14:0	84.04	75.06	71.79	3.55	0.032	0.011	0.331
C16:0	660.33	612.63	579.24	50.51	0.021	0.030	0.103
C16:1ω-7	70.99	60.21	63.02	4.45	0.192	0.432	0.229
C18:0	445.01	487.89	488.54	34.11	0.452	0.235	0.109
C18:1ω-9	559.58	573.21	616.74	45.00	0.042	0.048	0.147
C18:1 *t*11	60.55	81.81	85.21	6.17	0.030	0.023	0.367
CLA *cis*-9 *trans*-11	29.23	35.10	25.76	3.23	0.432	0.114	0.174
CLA *trans-*10 *cis-*12	27.93	24.03	32.33	2.41	0.565	0.210	0.189
C18:2ω-6	346.09	377.46	358.12	25.21	0.261	0.112	0.159
C18:3ω-3	18.53	36.18	53.70	3.00	0.019	0.012	0.213
C20:4ω-6	184.00	173.61	170.15	10.16	0.515	0.451	0.123
C20:5ω-3	30.28	47.52	58.91	4.22	0.040	0.013	0.479
C22:5ω-3	48.81	66.15	81.65	6.74	0.021	0.012	0.210
C22:6ω-3	43.33	53.46	49.32	6.29	0.452	0.229	0.341
∑SFA	1189.64	1175.85	1139.57	40.22	0.420	0.832	0.561
∑MUFA	691.39	742.23	765.56	22.11	0.231	0.553	0.319
∑PUFA	729.24	813.78	829.95	19.11	0.012	0.049	0.543
∑ω-3	140.94	203.31	243.59	0.76	0.012	0.027	0.345
∑ω-6	532.70	551.34	528.27	1.52	0.332	0.214	0.120
ω-6:ω-3	3.77	2.71	2.17	0.43	0.037	0.034	0.085
UFA:SFA	1.21	1.33	1.40	0.14	0.558	0.093	0.277
PUFA:SFA	0.62	0.69	0.73	0.08	0.088	0.093	0.193
Total FA	2610.27	2731.86	2740.23	25.75	0.285	0.444	0.195
Cholesterol	50.63	47.36	45.96	4.62	0.773	0.285	0.645

∑SFA = (C14:0 + C16:0 + C18:0), ∑MUFA = (C16:1+ C18:1+ C18:1 *trans*-11), ∑UFA = (C16:1+ C18:1+ C18:1 *trans*-11+ CLA *cis*-9 *trans*-11+ CLA *cis*-12 *trans*-10 + ∑ω-3 + ∑ω-6), ∑PUFA = (C18:1 *trans*-11+ CLA *cis*-9 *trans*-11+ CLA *cis*-12 *trans*-10 + ∑ω-3 + ∑ω-6), ∑ω-3 = (C18:3ω-3 + C20:5ω-3 + C22:5ω-3 + C22:6ω-3), ∑ω-6 = (C18:2ω-6 + C20:4ω-6). ω-6:ω-3 = (C18:2ω-6 + C20:4ω-6) ÷ (C18:3ω-3 + C20:5ω-3 + C22:5ω-3 + C22:6ω-3), UFA:SFA = (∑UFA)/∑SFA), PUFA:SFA = (∑PUFA/∑SFA). ^a^80 % canola oil and 20 % palm oil blend

**Table 3:** Effect of dietary treatments on proximate composition of *semimembranosus* muscle in goats

	Level of CPOB^a^, %				*P* value		
Parameter	0	4	8	SE	Overall	Linear	Quadratic
Moisture	72.27	72.43	71.78	0.88	0.8677	0.219	0.833
Protein	22.34	21.99	22.36	0.47	0.8309	0.308	0.190
Ether extract	4.12	4.07	4.39	0.68	0.9409	0.403	0.228
Ash	1.25	1.50	1.46	0.16	0.5478	0.114	0.330

^a^80 % canola oil and 20 % palm oil blend

The effect of dietary CPOB and postmortem ageing on physicochemical properties of SM muscle from goats is shown in Table 4. Diet did not affect (*P* > 0.05) muscle glycogen and pH at 45 min and 24 h postmortem. The muscle glycogen and pH observed at 24 h postmortem in all the treatments were significantly (*P <* 0.05) lower compared to those observed at 45 min postmortem. Diet had no effect (*P >* 0.05) on the drip loss and shear force values of SM muscle from goats. The drip loss increased (*P <* 0.05) while the shear force decreased (*P <* 0.05) as postmortem ageing advanced. Neither diet nor postmortem ageing affected (*P >* 0.05) cooking loss. The control meat had lower (*P <* 0.05) redness (a*) compared with other treatments on 1 and 4 d postmortem. Diet had no effect (*P >* 0.05) on a* value on 7 d postmortem. The lightness (L*) and yellowness (b*) were not influenced (*P >* 0.05) by diet. Postmortem ageing did not affect (*P >* 0.05) the L*, a* and b* values of SM muscle from goats.

**Table 4:** Effect of diet and postmortem storage on physicochemical properties of *semimembranosus* muscle in goats

		Level of CPOB^a^, %				*P* value	
Parameter	Time	0	4	8	SEM	Diet	Diet x time
Glycogen, mg/g	45 min	1.07^x^	1.06^x^	1.20^x^	0.14	0.549	0.231
	24 h	0.52^y^	0.54^y^	0.67^y^	0.07	0.166	
	*P* value	0.031	0.010	0.020			
pH	45 min	5.90^x^	5.95^x^	6.05^x^	0.07	0.474	0.129
	24 h	5.41^y^	5.45^y^	5.47^y^	0.07	0.892	
	*P* value	0.045	0.041	0.043			
Drip loss, %	1 d	4.31^x^	4.38^x^	4.32^x^	0.32	0.104	0.312
	4 d	7.92^y^	7.16^y^	6.91^y^	0.17	0.586	
	7 d	8.90^z^	7.93^z^	7.44^z^	0.16	0.445	
	*P* value	0.032	0.040	0.032			
Cooking loss, %	1 d	39.55	38.15	35.67	1.48	0.186	0.147
	4 d	36.98	36.68	35.17	1.44	0.303	
	7 d	32.36	35.01	35.14	0.85	0.411	
	*P* value	0.901	0.834	0.789			
	1 d	1.23^z^	1.32^x^	1.27^x^	0.40	0.703	0.451
Shear force, kg	4 d	0.96^y^	0.92^y^	0.98^y^	0.23	0.794	
	7 d	0.74^z^	0.74^z^	0.76^z^	0.17	0.234	
	*P * value	0.024	0.012	0.035			
Lightness (L*)	1 d	30.82	31.87	32.24	0.70	0.406	0.612
	4 d	33.57	32.58	32.55	1.27	0.817	
	7 d	31.49	30.41	33.09	1.26	0.365	
	*P* value	0.701	0.349	0.386			
Redness (a*)	1 d	12.59^b^	13.74^a^	14.66^a^	0.40	0.003	0.331
	4 d	12.05^b^	13.06^ab^	14.04^a^	0.54	0.041	
	7 d	12.04	12.38	12.79	0.90	0.948	
	*P* value	0.290	0.415	0.332			
Yellowness (b*)	1 d	14.36	11.87	12.84	0.77	0.129	0.212
	4 d	12.31	13.19	12.69	1.43	0.911	
	7 d	12.37	13.71	12.69	1.24	0.445	
	*P* value	0.347	0.124	0.330			

^a, b, c,^ means having different superscript along the same row are significantly different (*P* < 0.05). x, y, z means having different superscript along the same column are significantly different (*P* < 0.05). ^a^80 % canola oil and 20 % palm oil blend

The antioxidant contents and TBARS value of SM muscle from goats fed varying level of CPOB is shown in Table 5. The concentration of α and γ tocopherol in SM muscle increased (*P <* 0.05) with increasing level of CPOB. Diet did not affect (*P* > 0.05) total carotenoids, δ-tocopherol and glutathione peroxidase activity in SM muscle. Diet had no effect (*P >* 0.05) on meat TBARS value on 0, 1 and 4 d postmortem. On d 7 postmortem, the meat of goats fed 4 and 8 % CPOB had lower (*P <* 0.05) TBARS value compared with the control samples.

**Table 5:** Effect of diet and postmortem storage on antioxidants and TBARS values of *semimembranosus* muscle in goats

		Level of CPOB^a^, %				*P* value	
Parameter	Time	0	4	8	SE	Diet	Diet x time
	0 d	2.43^bx^	3.66^ax^	4.39^ax^	0.54	0.026	0.476
α-tocopherol, mg/kg	1 d	2.40^bx^	3.33^ay^	4.20^ax^	0.34	0.034	
	4 d	2.36^bx^	3.05^aby^	3.91^ax^	0.34	0.024	
	7 d	2.00^by^	2.76^ay^	3.10^ay^	0.32	0.395	
	*P* value	0.012	0.022	0.031			
	0	0.70^bx^	0.80^ax^	0.83^ax^	0.08	0.048	0.614
γ-tocopherol, mg/kg	1	0.67^by^	0.79^ax^	0.80^ax^	0.05	0.045	
	4	0.65^ay^	0.73^ay^	0.73^ay^	0.03	0.001	
	7	0.65^ay^	0.68^ay^	0.70^ay^	0.07	0.272	
	*P* value	0.010	0.042	0.044			
	0	0.09^x^	0.08^x^	0.11^x^	0.01	0.530	0.513
δ-tocopherol, mg/kg	1	0.07^x^	0.08^x^	0.09^xy^	0.03	0.058	
	4	0.05^y^	0.05^y^	0.07^y^	0.16	0.411	
	7	0.04^y^	0.05^y^	0.07^y^	0.01	0.164	
	*P* value	0.001	0.003	0.003			
	0	0.14^x^	0.27^x^	0.38^x^	0.04	0.053	0.231
	1	0.11^y^	0.19^y^	0.22^y^	0.05	0.051	
Total carotenoids, mg/kg	4	0.11^y^	0.15^y^	0.14^y^	0.03	0.053	
	7	0.11^y^	0.14^y^	0.14^y^	0.08	0.176	
	*P* value	0.043	0.022	0.019			
	0	81.41	64.77	60.33	13.71	0.052	0.134
	1	86.55	67.44	62.97	12.44	0.055	
GPX activity^b^	4	86.76	57.05	50.21	13.08	0.192	
	7	87.36	76.76	66.56	7.25	0.149	
	*P* value	0.187	0.901	0.782			
	0	0.12^x^	0.10^x^	0.08^x^	0.11	0.466	0.612
TBARS, mg MDA/kg	1	0.25^x^	0.26^x^	0.24^y^	0.15	0.264	
	4	0.42^xy^	0.40^x^	0.34^y^	0.25	0.393	
	7	0.85^ay^	0.64^by^	0.56^by^	0.21	0.047	
	*P* value	0.030	0.011	0.023			

^a, b, c,^ means having different superscript along the same row are significantly different (*P* < 0.05). x, y, z means having different superscript along the same column are significantly different (*P* < 0.05). ^b^Glutathione peroxidase activity is expressed as nmoles NADPH oxidized /min/mg protein. ^a^80 % canola oil and 20 % palm oil blend

**Fig. 1:**
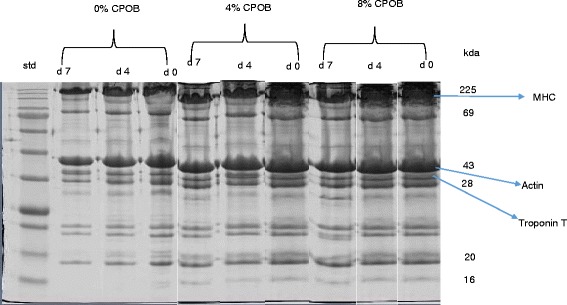
SDS-PAGE of myofibrillar proteins of *Semimembranosus* muscle from goats. Equal amounts of protein (30 μg) of each sample was loaded and electrophoresed on a separate 12 % sodium dodecyl sulfate polyacrylamide gel electrophoresis (SDS-PAGE) at a constant voltage (120 V) for 90 min. The gels were stained with Coomassie blue staining for 60 min and destained with destaining solution for 45 min. d0 = day 0, d4 = day 4, d7 = day 7. CPOB = 80 % canola oil and 20 % palm oil blend. Std = standard. MHC = myosin heavy chain

The concentration of carbonyl, free thiol, myosin heavy chain, actin and troponin T in the SM muscle from goats is shown in Table 6. The SDS-PAGE pattern of myofibrillar proteins of SM muscle is shown in Fig. 1. Diet had no effect (*P >* 0.05) on the concentration of free thiol, carbonyl, actin, troponin T and myosin heavy chain throughout storage. The concentration of free thiol, myosin heavy chain and troponin T decreased (*P <* 0.05) while the carbonyl content increased (*P <* 0.05) as storage progressed. The concentration of actin remain unchanged (*P >* 0.05) throughout storage.

**Table 6:** Effect of diet and postmortem storage on protein oxidation and myofibrillar protein profile of *semimembranosus* muscles in goats

		Level of CPOB^a^, %				*P* value	
Parameter	Time	0	4	8	SE	Diet	Diet × time
Free thiol, nmol/mg	0 d	55.21^x^	54.99^x^	55.87^x^	5.62	0.210	0.332
	4 d	47.19^y^	48.22^y^	48.89^y^	4.66	0.561	
	7 d	42.11^z^	43.00^z^	43.67^z^	3.99	0.215	
	*P* value	0.01	0.01	0.01			
Carbonyl, nmol/mg	0	1.59^x^	1.62^x^	1.59^x^	0.20	0.112	0.445
	4	2.60^y^	2.57^y^	2.57^y^	0.32	0.123	
	7	4.09^z^	4.00^z^	3.97^z^	0.38	0.167	
	*P* value	0.02	0.01	0.01			
Myosin heavy chain, mg	0	24.34^x^	25.02^x^	25.00^x^	3.12	0.542	0.715
	4	20.22^y^	21.67^y^	22.00^y^	3.00	0.123	
	7	17.19^z^	18.00^z^	18.99^z^	2.19	0.334	
	*P* value	0.02	0.03	0.01			
Actin, mg	0	8.22	8.18	8.50	1.20	0.145	0.319
	4	8.12	8.10	8.41	1.20	0.332	
	7	8.00	8.00	8.22	1.09	0.212	
	*P* value	0.12	0.45	0.52			
Troponin T, mg	0	2.45^x^	2.60^x^	2.59^x^	0.25	0.09	0.092
	4	2.00^y^	2.21^y^	2.19^y^	0.29	0.08	
	7	1.65^z^	1.90^z^	2.00^z^	0.31	0.08	
	*P* value	0.01	0.01	0.01			

x, y, z means having different superscript along the same column are significantly different (*P* < 0.05). ^a^80 % canola oil and 20 % palm oil blend

## Discussion

The FA profile of SM muscle from goats was amenable to dietary CPOB. Dietary supplementation of CPOB in goats’ diet decreased the concentration of C14:0 and C16:0 in SM muscle. The finding could be due to the decline in the mRNA abundance and the activity of lipogenic enzymes such as fatty acid synthase and acetylCoA carboxylase required for the synthesis of medium chain fatty acids [8, 40, 41]. This finding is consistent with those of Loor et al. [41] who observed that the supplementation of 3.3 % canola oil, canolamide or canola oil-canolamide mix reduced the concentration of C14:0 and C16:0 in bovine milk [41]. Similarly, Najafi et al. [42] observed that the *longissimus lumborum* muscle from Mahabadi goats fed 2 % soybean oil had lower concentration of C14:0 and C16:0 compared with those fed palm oil or fish oil. The increase in the concentration of C18:1ω-9 in the tissue of oil-fed goats could be attributed to the higher dietary intake of C18:1ω-9 [43] or the delta-9 desaturation of C18:0 in the tissue. This finding is consistent with the results of a companion *in vitro* trial [44] which showed that the concentration of C18:1ω-9 after 24 h incubation increased as the level of CPOB increased in the substrate. The C18:1 *trans*-11 is a mutual intermediate product of biohydrogenation of unsaturated 18-carbon fatty acids [8, 40]. Consequently, the significant increase in the concentration of C18:1 *trans*-11 in the SM muscle from goats fed 4 and 8 % CPOB could be due to the higher total unsaturated fatty acids in the diets compared with that of the control goats. This observation corroborates the findings of Loor et al. [41] who observed that the supplementation of 3.3 % canola oil, canolamide or canola oil-canolamide mix increased the concentration of C18:1 *trans*-11 and C18:1ω-9 in bovine milk. The linear increase in the concentration of C18:3ω-3 with incremental level of CPOB was expected given the increase in its intake [43] and its concentration in the diets. The increase in the concentration of C20:5ω-3 and C22:5ω-3 in goats fed 4 and 8 % CPOB compared to the control goats, mirrored the increase in muscle C18:3ω-3. The long chain n-3 PUFA are metabolites of C18:3ω-3 [40]. As found in the current study, dietary C18:3ω-3 led to a significant increase in muscle C18:3ω-3 and long chain n-3 PUFA in lambs [40]. In contrast, the supplementation of 3 % canola oil to goats [45] and 7.4 % linseed oil to lamb [8] did not increase the concentration of the long chain ω-3 PUFA despite the increase in the concentration of C18:3ω-3. The PUFA/SFA ratio for all the treatments was beneficially higher than the recommended value (>0.4) indicating the healthfulness of goat meat compared with other ruminant species [46]. Although, dietary CPOB lowered the ω-6/ω-3 in the SM muscle, the ω-6/ω-3 for all treatments was within the normal range (<4) recommended for healthy diet [47]. Dietary CPOB had no effect on the concentration of cholesterol in the SM muscle from goats. This observation is in tandem with the report of Oliveira et al. [48] who observed that the cholesterol content of *longissimus dorsi* muscle from Nellore steers fed different oils was similar to those fed the control diet. Diet had no effect on the chemical composition of SM muscle from goats. This observation could be attributed to the similar growth rate and slaughter weight of the goats [43]. This finding concurs with those of Marinova et al. [49] who observed that dietary sunflower oil had no effect on the proximate composition of mutton. Similarly, Najafi et al. [42] did not observe changes in the chemical composition of Mahabadi goats fed 2 % palm oil, soybean oil or fish oil.

Dietary CPOB had no effect on muscle glycogen and pH. This observation could be due to the similar metabolizable energy of the diets and the homogenous management and slaughter conditions employed in the course of the feeding trial [43]. The muscle glycogen and pH declined at 24 h postmortem. This could be due to postmortem glycolysis [32, 50]. Similarly, oil supplementation did not affect muscle pH in goats [43] and beef cattle [48]. The ultimate pH (pH_24_) of meat samples in all the treatments was within normal pH range reported for goats [46].

Water holding capacity is an important meat quality attribute [51]. Dietary CPOB had no effect on the drip loss of *semimembranosus* muscle in goats. This observation could be due to the similarity in protein oxidation (loss of thiol, decrease in myosin heavy chain and troponin T and increase in carbonyl content) across dietary treatments. Oxidation of myofibrillar proteins can affect the functionality of the muscle proteins thereby reducing the water holding capacity of the muscle [52]. The increase in drip loss over storage could be due to the increase in protein oxidation or the structural breakdown of muscle fibers which reduce their ability to hold water [52]. This finding is consistent with those of Sabow et al. [53] who observed an increase in drip loss during postmortem ageing of chevon. Dietary CPOB had no effect on cooking loss and shear force value of SM muscle from goats. This observation could be due to the similar intramuscular fat content of the SM muscle from the goats. Increase in Intramuscular fat content reduces cooking loss and shear force value of meat [52]. Similarly, the dietary supplementation of different oils did not affect the cooking loss and shear force of *longissimus dorsi* muscle from goats [42]. The decrease in shear force as postmortem ageing advanced could be due to the degradation of myofibrillar proteins. Similar finding was observed in chevon aged for 14 d [30, 53].

Color is an important meat quality trait because the first impression consumers have on any meat product is based on its color [51]. Dietary CPOB enhanced the a* value of SM muscle from goats. Ponnampalam et al. [54] posited that the antioxidant status of muscle at slaughter is the major factor that determines color stability and redness of meat. Thus, the significant increase in the concentration of α and γ tocopherol in the muscle of goats fed 4 and 8 % CPOB justifies the higher a* value of their meat. The current observation contradicts findings in goats [43] and cattle [48] in which meat color was not altered following dietary supplementation of different oils. However, the current finding supports the report of Jensen et al. [55] who observed that dietary rapeseed oil enhanced a* value in pork compared to the control diet. Since differences in lipid oxidation could not be established between the treatments on d 1 and 4 postmortem, it is probable that the increase in a* value observed on d 1 and 4 postmortem in the meat of goats fed 4 and 8 % CPOB resulted from the decrease in the conversion of red myoglobin to brown metmyoglobin. However, this contention warrants further investigation. Decreased myoglobin oxidation was observed in beef following dietary supplementation of vitamin E [56].

The significant increase in the concentration of α and γ tocopherol with the increase in the level of CPOB mirrored the antioxidants contents of the diets. Similarly, an increase in muscle vitamin E was observed in pigs fed canola oil [57] and rapeseed oil [55]. Both tocopherol and carotenoids are fat soluble vitamins. Thus, the higher fat content in the 4 and 8 % CPOB diets compared with the control diet might have enhanced the absorption of the antioxidant vitamins in the gastrointestinal tract and their subsequent deposition in the muscle.

Antioxidant enzymes constitute an intracellular barrier against free radicals and their activity in vivo is modulated by various factors [9]. However, after slaughtering an animal, all cells will be in anoxia and depleted of nutrients [58]. In this conditions enzyme activity can be regarded only to the remnant at the onset of cell death [9, 58, 59]. Thus, the behavior of antioxidant enzymes during postmortem depends on the individual redox status prior slaughter [9, 58, 59]. The similarity in the GPX activity across the treatments is an indication that dietary CPOB did not cause oxidative deteriorations in chevon in spite of the increase in the n-3 FA. Feeding oils high in PUFA has been shown to increase postmortem GPX activity [9, 58] in response to the need to curb lipid and protein oxidation. A negative relationship was observed between tissue α-tocopherol levels and GPX activity, and a positive relationship was evinced between GPX activity and TBARS [58]. The current observation could be due to the increase in the concentration of α and γ tocopherol in the SM muscle from goats fed 4 and 8 % BCPO compared with those fed the control diet. Maraschiello et al. [58] observed that the meat of chicken fed vitamin E had higher α-tocopherol content and lower GPX activity compared to the non-supplemented chickens. In contrast, Renerre et al. [9] reported that dietary vitamin E had no effect on GPX activity in turkey meat. Vitamin E is located in the membrane where it efficiently functions to protect highly oxidizable PUFA from oxidation [59]. Thus, a higher tocopherol content in the membrane would enhance oxidative stability thereby lowering the requirement for GPX [59].

The concentration of α, γ and δ-tocopherol and total carotenoids decreased as postmortem storage progressed. This reflects the breakdown of antioxidant defense system. The GPX activity was stable throughout the 7 d postmortem storage. In line with the current observation, Renerre et al. [60] espoused the stability of GPX during an 8 d refrigerated storage of beef.

Ponnampalam et al. [10] observed that lamb muscle having vitamin E content lower than 2.95 mg/kg muscle was susceptible to PUFA oxidation. The higher TBARS value in the control meat on d 7 postmortem could be due to its lower α-tocopherol content compared with other treatments. The increase (*P <* 0.05) in TBARS values as postmortem storage progressed could be attributed to the decrease in the concentration of α, γ, and δ-tocopherol and total carotenoids. The increase in TBARS over storage is consistent with findings in beef [60], chevon [30, 61], broiler meat [58, 62] and turkey meat [9].

Free thiol and carbonyl contents are important indicators of protein oxidation in foods [9, 53]. Dietary CPOB did not affect the free thiol and carbonyl contents throughout storage. This suggests that the increase in α and γ tocopherol in the meat of the oil-fed goats was not sufficient to curb protein oxidation. The decrease in free thiol during ageing is consistent with the findings of Sabow et al. [53] who observed a decrease in free thiol content during postmortem ageing of chevon for 14 d. Renerre et al. [9] observed that carbonyl content of turkey meat increased during a 9 d chill storage. Diet had no effect on the concentration and SDS-PAGE expression of myofibrillar proteins of semimembranosus muscle from goats. Myosin heavy chain (MHC) is the most abundant myofibrillar protein in skeletal muscle of animals and it is readily susceptible to oxidation [26, 53]. The concentration of MHC and troponin T decreased as storage progressed while actin was stable throughout storage. This finding is consistent with those of Sabow et al. [30] who observed a significant decrease in the band intensity of MHC and troponin T and a non-significant decrease in actin during a 14 d postmortem ageing of goat meat.

## Conclusions

The results of this study evinced that dietary CPOB when fed up to 8 % DM can beneficially alter the lipid profile of *Semimembranosus* muscle from goats to meet contemporary nutritional and health guidelines. Also, CPOB enhanced the tocopherol content and meat redness and reduced lipid oxidation in goat meat. Dietary BCPO had no effect on protein oxidation and myofibrillar protein profile of goat meat. Further studies to determine the effect of CPOB on oxidative stability of myoglobin in *semimembranosus* muscle in goats is suggested. In addition, the impact of dietary CPOB on lipid composition and physicochemical properties of other muscles should be examined.
